# Enhanced Combustion Properties of Al-Si Eutectic Alloy in Energetic Mixtures

**DOI:** 10.3390/ma17194890

**Published:** 2024-10-05

**Authors:** Tlek Ketegenov, Igor Vongay, Oksana Chervyakova, Bakhyt Kalmuratova, Anton Kononov, Kaster Kamunur, Rashid Nadirov

**Affiliations:** 1 Institute of Combustion Problems, Bogenbay batyr street 172, Almaty 050012, Kazakhstan; tlek58@mail.ru (T.K.); konan_hitech@mail.ru (A.K.); nadirov.rashid@gmail.com (R.N.); 2 LLP “AlmaDK”, Almaty 041605, Kazakhstan; vongaiicp@mail.ru (I.V.); oksana_almadk@mail.ru (O.C.); 3 Faculty of Chemistry and Chemical Technology, Al-Farabi Kazakh National University, Almaty 050040, Kazakhstan; bakhyt.kalmuratova@mail.ru

**Keywords:** aluminum powder, Al-Si eutectic alloy, energetic mixture, burning rate, thermal decomposition

## Abstract

This study investigates the feasibility of using an Al-Si eutectic alloy as a reactive fuel in energetic mixtures. Al-Si eutectic alloy powders were obtained from secondary resources and ground to a particle size of less than 100 μm. We examined these powders’ burning characteristics and thermal properties compared to pure Al powder. Results showed that the burning rate of energetic mixtures containing Al-Si eutectic alloys was 1.5 to 2.0 mm/s higher than those with pure Al. Additionally, the activation energy of pure PTFE was reduced from 81.29 kJ/mol to 61.75 kJ/mol when the Al-Si alloy was added. The formation of oxides, carbides, and fluorides in the combustion products of Al-Si-based mixtures significantly influenced their thermodynamics.

## 1. Introduction

Aluminum powder with a high energy density is used in solid propellants, explosives, pyrotechnic compounds, etc., and is widely used as a reactive fuel in energetic mixtures [1,2,3,4,5,6]. However, the surface layer of aluminum powder is easily oxidized during preparation, storage, and use, forming oxide films. Formed metal oxide films directly affect its ignition and burning indicators [7]. Also, the process of the agglomeration of particles during the burning of aluminum powder limits the possibility of its use [8]. In addition, the particle size, burning characteristics, and thermodynamic properties of components significantly impact the preparation of energetic fuels [9]. The particle sizes of aluminum powder used as reactive fuel in energetic mixtures vary from nano- to submicrometer [10,11]. Many research works have shown that the technology for obtaining aluminum powder from metallic aluminum is carried out using several processes [12,13]. In particular, metallic aluminum is melted in an inert environment, centrifugal atomization is performed, and aluminum powder particles are collected. However, since these processes occur at high temperatures, in an inert environment, and at high rotational speeds of the centrifuge, the production cost of aluminum powder increases significantly.

To compensate for some of the shortcomings of pure aluminum powder, currently, the modification of aluminum powder [14,15] or different aluminum alloys (in particular, Al-Mg alloy [16,17], Al-Ti alloy [18], Al-Ni alloy [19,20], Al-Li alloy [21,22,23], etc.) have been widely studied for their use in solid propellants. Compared to pure aluminum, these alloys are characterized by a lower ignition temperature, lower density, a higher burning temperature, more formation of gas-phase products, and the complete combustion of fuels [24,25]. Based on these advantages, using alloys as reactive fuel can influence energetic mixtures’ thermodynamic properties and kinetic performance, prompting continued research into aluminum-based alloys. Ao et al. [26] studied the sintering and burning characteristics of various aluminum-based alloys used as fuel in solid propellants. In comparison, the heat of combustion and the burning rate of the Al-Si alloy are higher than those of aluminum and other aluminum alloys. Therefore, Al-Si alloy powder was compared with pure aluminum powder in this research. In our research, several main criteria were used to make the choice of the Al-Si alloy: first, the high degree of hardness of the Al-Si eutectic alloy; second, the Al-Si alloy is a secondary resource; third, solid fuel has a high burning rate and a high specific heat impulse (ISP). Therefore, the Al-Si alloy required further research [27,28,29,30].

One of the most critical aspects of Al-Si eutectic alloy performance is its thermal decomposition property, which significantly affects burning parameters. This process involves the thermal decomposition of the silicon component and oxidation of aluminum at high temperatures, increasing the energy release rate during burning for Al-Si eutectic alloys. Silicon modifies the oxide layer of aluminum and facilitates the efficient oxidation of aluminum by lowering the temperature required for burning [31,32]. In addition, the thermal decomposition of the Al-Si eutectic alloy results in improved ignition characteristics and a higher burning rate than pure aluminum. When the alloy is heated, the eutectic structure melts at a low temperature (577 °C), which allows the reactive fuel to transition to the liquid phase more quickly. This phase transition leads to accelerated oxidation kinetics of silicon and improved thermal conductivity. These properties allow the Al-Si eutectic alloy to be used as a reactive fuel in energetic mixtures where rapid energy release and fuel efficiency are key performance indicators [33,34].

Al/PTFE-based energetic mixtures have been widely studied to evaluate the thermodynamic properties of aluminum powder [35,36]. The high thermal properties of the chemical reaction between the components make it more effective than other studied energy additives. Therefore, to evaluate the burning characteristics and thermal properties of the Al-Si eutectic alloy, energetic mixtures based on Al-Si alloy–polytetrafluoroethylene (Al-Si/PFTE) or Al/PTFE systems were prepared and compared.

The main goal of this study is to prepare Al-Si alloy powder from secondary materials using mechanical methods, which can be used as a reactive fuel in laboratory conditions. A comparison of the prepared Al-Si alloy powder’s composition, morphology, and thermal properties with pure aluminum powder is also a goal of this study. In addition, the burning characteristics and thermodynamic properties of the Al-Si eutectic alloy as a reactive fuel in energetic mixtures are used as a comparison study with pure aluminum powder.

## 2. Materials and Methods 

### 2.1. Materials

Aluminum powder (Houston, TX, USA) is produced by spraying primary aluminum or its waste. The percentage of active aluminum is 99%. As for the Al-Si eutectic alloy, secondary resources were obtained and prepared by grinding with special equipment. Polytetrafluoroethylene (Teflon, fluoroplastic-4, F-42) (-C2F4-) (Sigma-Aldrich, Germany 468096) was used as an oxidizer and binder.

### 2.2. Research Instruments

The morphological images and elemental composition of the initial Al and Al-Si alloy powder samples were determined using a Quanta 200i 3D (FEI Company, Hillsboro, OR, USA), Scanning Electron Microscope (SEM), and Energy Dispersive X-ray Spectroscope (EDX). The phase composition of the propellant powder and energetic mixture burning products was determined using a Bruker D8 Advance diffractometer (Billerica, MA, USA) with CuKα (40 kV, 40 mA) radiation.

### 2.3. Measurement of Thermal Properties of Initial Components and Energetic Mixtures

Thermal analysis studies the kinetics of the thermal decomposition of initial components and energetic mixtures, and it is an effective method for calculating the activation energy. The thermal decomposition of each sample was investigated using a BAXIT thermogravimetric analyzer (VHT-TGA-103) in a nitrogen atmosphere, in a temperature range of 25–1200 °C at different heating rates (5, 10, 15, and 20 °C/min). Each initial component and energetic mixture was measured three times in thermogravimetric analyzer equipment, and the average was used for analysis.

### 2.4. Preparation of Al-Si Alloy-Based Powders

**Figure 1:**
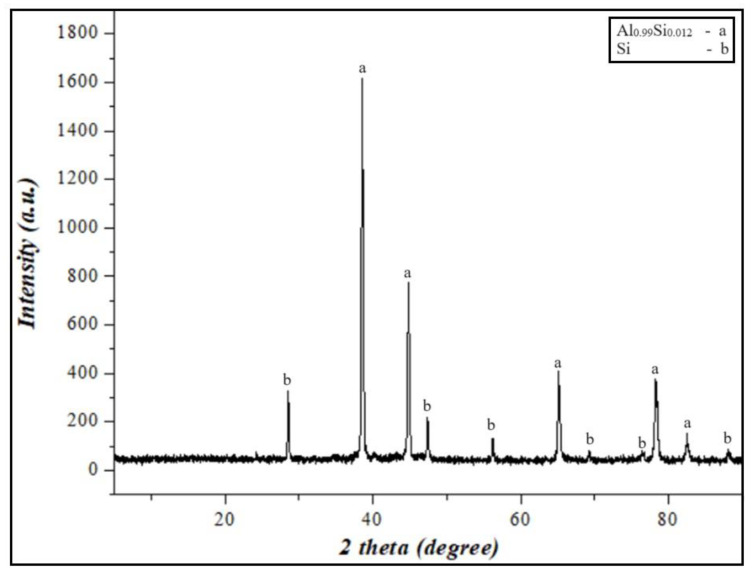
XRD results of Al-12%Si alloy powder.

Secondary resources based on Al-Si alloys were crushed using a unique laboratory jaw crusher (ShD-10 Jaw crusher). Each ground size was subjected to sieve analysis. In addition, the Al-Si powder from the crusher was further ground using a laboratory disintegrator. The powder obtained from the disintegrator was ground in a ball mill for 5 min to a size of 100 μm > d and separated using a vibrating sieve. The results of the compositional analysis of the obtained powders are given in Figure 1. Structural studies were carried out, and the results of the study are presented in Section 3.2.

### 2.5. Preparation of PTFE/Al-Si-Based Energetic Mixtures

Polymer tetrafluoroethylene (PTFE) was used as an oxidizer in energy mixtures. Pure aluminum powder or Al-Si alloy powder was compared for use as a reactive fuel. Al-Si alloy powder with particle diameters below 100 μm in different ratios with PTFE powder (PTFE 75%/Al-Si; PTFE 50%/Al-Si; PTFE 25%/Al-Si; PTFE 10%/Al-Si) in ball mills was mixed. Cylindrical samples with a diameter of 14.85 mm and a height of 20 mm were prepared by pressing with a force of 100 kN in special press equipment. This initiates the sample with a sphere by sending a current with a voltage of 20V through the current source. A high-speed camera videotaped the combustion process of the fuel mixture, and the burning rate was calculated. The ignition process was repeated 3 times for each sample.

## 3. Results and Discussion

### 3.1. Preparation of Al-Si Alloy Powder

Due to its elastic and malleable properties, extracting aluminum by mechanical processes is difficult in the preparation of aluminum powder from metallic aluminum. Therefore, it was obtained only by melting and dusting aluminum powder [37,38]. In comparison, the Al-Si alloy was ground from secondary resources based on mechanical methods. Two main parameters for using these methods were the mechanical stiffness of the relative secondary resources and the cubic and diamond-like crystal lattice of silicon atoms. Therefore, mechanical methods were considered more effective in preparing Al-Si alloy powder from secondary resources. Secondary resources were crushed using a jaw crusher and a laboratory disintegrator and classified into fractions using vibrating sieves. Different fractions were ground in a laboratory ball mill, and particles with a size of 100 μm > d were separated using a vibrating sieve. Powdered Al-Si alloy compositional and structural analyses were carried out, and the results are discussed in Section 3.2.

### 3.2. Morphology and Composition of Al and Al-Si-Based Alloy Fuels

XRD analyses of the Al-Si-based alloy prepared by grinding were performed. As a result, aluminum silicon (Al_0.99_Si_0.012_) and silicon (Si) were identified as crystalline phases in the Al-Si alloy powder, and they are presented in Figure 1. The results showed that the mass fraction of silicon concentration in the secondary resources of aluminum was 12.1%. According to the phase diagram of the Al-Si alloy [39], the solubility of silicon in aluminum is 12.6%. Therefore, the secondary aluminum resources obtained for research work can be considered as an Al-Si eutectic alloy.

**Figure 2:**
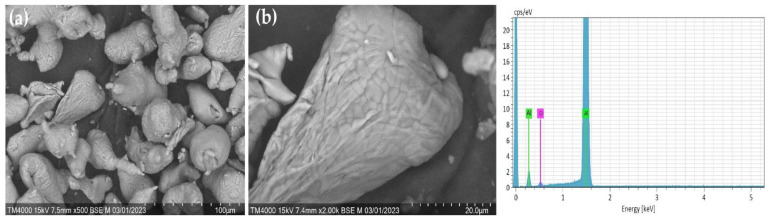
SEM and EDX analysis results of the initial pure Al powders. (**a**) Al particles; (**b**) Thin aluminum oxide film.

**Figure 3:**
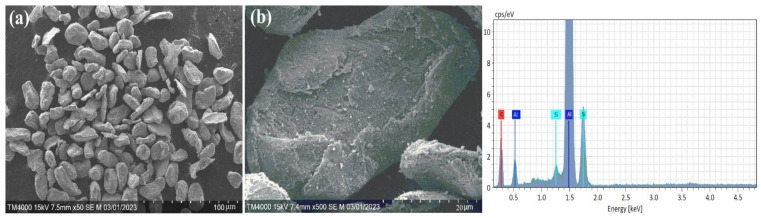
SEM and EDX analysis results of the Al-Si alloy powders. (**a**) deformed Al-Si particles; (**b**) cracked Al-Si particles.

In addition, Figure 2 shows the morphology and compositional analysis results of the original pure Al powder. The different shapes and complex surface structures of the aluminum particles in Figure 2a indicate that the powder was obtained mechanically or by other complex manufacturing methods. Such particle shapes can have high reactivity or good mechanical properties. However, as shown in Figure 2b, the aluminum particles are covered by a thin aluminum oxide film. In addition, the EDX results indicate that aluminum contains a surface oxide layer. This may result from the natural oxidation of aluminum, as aluminum powder oxidizes quickly with air. In comparison, the morphology of mechanically prepared Al-Si alloy powder is shown in Figure 3. The structure of the Al-Si alloy is more bent, deformed (Figure 3a), and cracked (Figure 3b) than pure aluminum powder. In addition, the XRD results (Figure 1) and EDX showed that no oxide composition was detected in the Al-Si alloy powder. It can be assumed that the absence of an oxide film on an Al-Si alloy allows for solving some of the shortcomings of pure aluminum particles (slow ignition, particle agglomeration, etc.) with Al-Si alloy powder.

**Figure 4:**
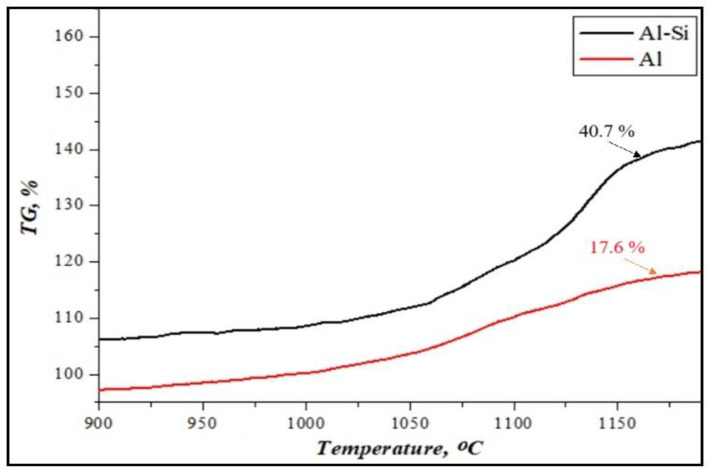
The result of thermogravimetric analysis of the weight gain of reactive fuels.

Figure 4 shows the mass change in the results of the thermogravimetric analysis of initial Al or Al-Si alloy-based reactive fuels at a heating rate of 10 °C/min.

It was determined that the oxidation process of fuels based on Al or Al-Si alloys takes place through exothermic reactions in the air atmosphere, using a thermogravimetric analyzer at a temperature of 900–1200 °C [40]. It is shown that the intensity of the weight gain of aluminum powder is not significantly higher (+17%). However, the weight gain of Al-Si alloys increased by up to 40%. It has been shown that adding silicon to the alloy’s composition can enhance aluminum’s oxidation reaction. This is because in Al-Si alloys, under the influence of silicon, deeper oxidation processes of aluminum particles may occur.

Calculating the activation energy (*E_a_*) is very important due to the thermal decomposition properties of initial components and energetic mixtures. Kissinger’s method is often used because the knowledge of the reaction order or reaction models is not considered necessary in calculating *E_a_*. Many researchers [41,42,43] have studied the activation energy of high-energy density materials and composite mixtures using the Kissinger method. Therefore, in this research work, the Kissinger and Ozawa methods are valuable tools for estimating the *E_a_* value of initial components and energetic mixtures.

The following equation is recommended in the calculation of *E_a,_* according to the Kissinger method:

(1) $$\frac{E_{a}}{R}=\frac{dln(\beta T_{p}^{-2})}{{dT}_{p}^{-1}}$$

where *E_a_* is the activation energy; *T_p_* is the highest temperature of thermal decomposition of the DTA curve; *R* is the universal gas constant; and *β* is the heating rate.

**Figure 5:**
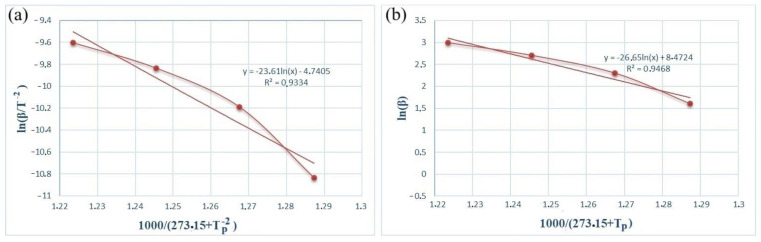
Kissinger (**a**) and Ozawa (**b**) plots of an PTFE/Al energetic mixture.

**Figure 6:**
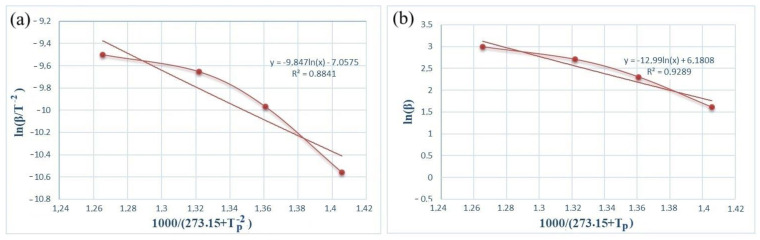
Kissinger (**a**) and Ozawa (**b**) plots of an PTFE/Al-Si energetic mixture.

Figure 5 and Figure 6 show the dependence graphs of the values of *ln(β/*$T_{p}^{-2}$) and $T_{p}^{-1}$ of the Kissinger (a) and Ozawa (b) methods for calculating the activation energy of energetic mixtures prepared based on Al and Al-Si alloy powders. These relationships show almost straight lines for each of the energetic mixtures. The numerical data on the graph were used to calculate Equation (1), which calculates the activation energies of each energetic mixture. The *E_a_* values calculated by the Kissinger method were defined as 156.71 and 61.75 kJ/mol for the Al and Al-Si alloy powders, respectively. The *E_a_* values calculated by the Ozawa method were 168.11 and 77.33 kJ/mol for the Al and Al-Si alloy powders, respectively. In comparison, the *E_a_* values calculated by different methods were very approximate. However, the *E_a_* values of the energetic mixture prepared with the Al-Si alloy powder were significantly lower than that of the Al powder.

At different heating rates, activation energies of reactive fuels and energetic mixtures were calculated using Kissinger’s (*E_k_*) and Ozawa’s (*E_a_*) methods. The calculated results are shown in Table 1.

The activation energy results calculated by the Kissinger and Ozawa methods showed relatively close values. The activation energy of pure aluminum powder was much higher than that of Al-Si alloy powders. This indicates that silicon in the alloy lowers the energy threshold required for primary ignition in jet fuels. It can be assumed that the oxide film on the surface of pure aluminum particles significantly increases the thermal degradation temperature. The cracks and deformations that occur during the mechanical grinding of secondary materials accelerate the chemical reaction. In addition, adding pure aluminum powder to PTFE did not significantly change the thermal degradation values. However, with the addition of the Al-Si alloy, the thermal decomposition value of PTFE was brought down from 480–550 °C to 440–520 °C. This is because it can be concluded that the advantages of the above Al-Si alloy significantly affect the activation energy of the energy mixture.

### 3.3. Application of Al-Si Alloy Reactive Fuels in Energetic Mixture

**Figure 7:**
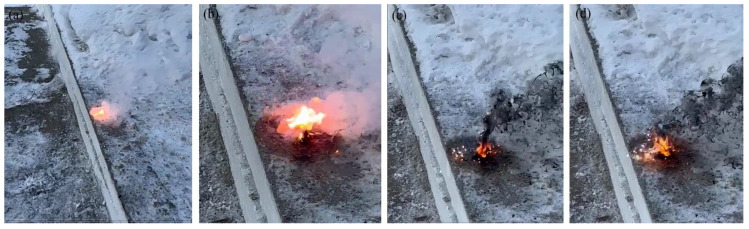
Burning cinegram of PTFE/Al-based energetic mixtures. (**a**) the beginning of burning; (**b**) development burning; (**c**) the climax of the burning; (**d**) complete combustion.

**Figure 8:**
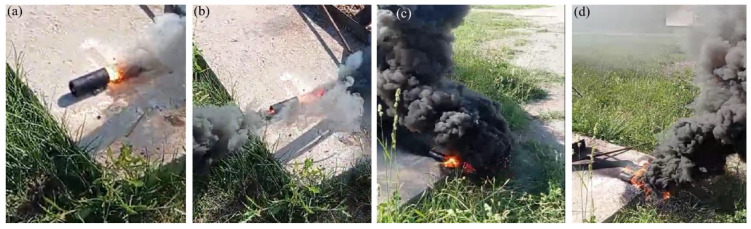
Burning cinegram of PTFE/Al-Si-based energetic mixtures. (**a**) the beginning of burning; (**b**) development burning; (**c**) the climax of the burning; (**d**) complete combustion.

The heat of combustion can describe the energy level of energetic mixtures. In addition, burning rate and activation energy are essential parameters that evaluate the combustion performance of energetic mixtures [44,45]. To evaluate the burning characteristics and thermal properties of the Al-Si alloy-based reactive fuel, the burning rate and activation energy of energy mixtures based on PTFE/Al and PTFE/Al-Si were comparatively studied in the atmospheric environment. Figure 7 and Figure 8 show the burning kinetics of energetic mixtures prepared using various fuels and PTFE. The figures show the beginning, development, climax, and complete combustion of the burning process from Figure 7a–d and Figure 8a–d.

According to the burning characteristics of energetic mixtures based on PTFE/Al, flashes are observed when large amounts of soot and teflon pyrolysis products are formed. In comparison, using Al-Si alloy powder as fuel was characterized by releasing a significant amount of carbon in the form of soot and a stable combustion with a self-supporting combustion wave front. In addition, the PTFE/Al-Si system was found to have a higher ignition temperature and a faster transition from combustion to detonation. Therefore, it can be assumed that energetic mixtures based on PTFE/Al-Si can be used in signal pyrotechnics.

**Figure 9:**
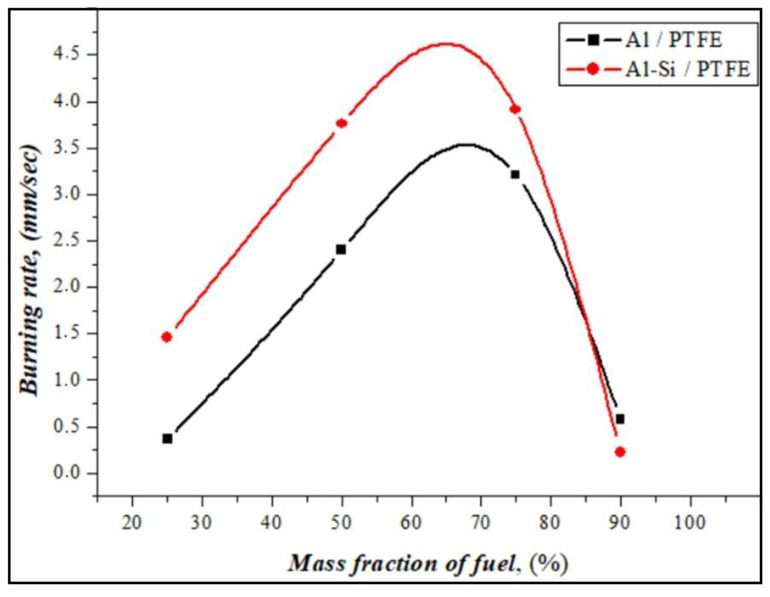
Experimentally measured burning rate of Al and Al-Si alloy fuels in energetic mixtures.

The burning rates of energetic mixtures prepared from Al or Al-Si alloy powders in the atmospheric environment were calculated according to the cinegram of burning, and the results are presented in Figure 9.

**Figure 10:**
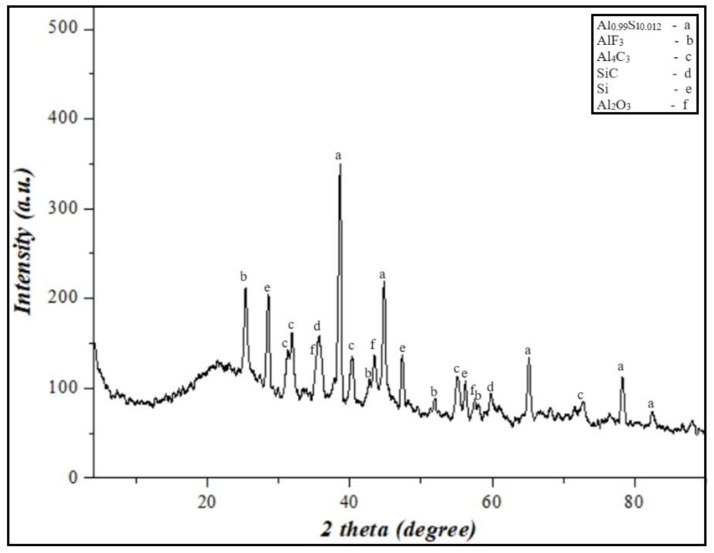
XRD results of the final products from the burning of PTFE/Al-Si 50/50 wt% energetic mixture.

At a low amount of reactive fuels, the burning rate with a large amount of soot formation during the combustion of energetic mixtures had a significantly low value. When the ratio of oxidizer and fuel was equalized (PTFE50%/Al-Si), the burning speed increased to 4 mm/sec with a stable combustion front. Samples with a ratio of PTFE25%/Al-Si burn intensely and quickly, and the amount of soot formation is significantly reduced. PTFE75%/Al-Si and PTFE10%/Al-Si have little or no burning. It has been shown that silumin ignites quickly and has a slightly higher burning rate than aluminum powders. Reactive fuel ignites quickly under the influence of the silicon in silumin and burns steadily. Figure 10 presents the results of the compositional analysis of solid combustion products based on chemical reactions between the fuel and oxidizer.

According to the results of the XRD, the presence of Al_2_O_3_ Al_4_C_3_, SiC, and AlF_3_ phases in the combustion products, exothermic oxidation-reduction reactions during the combustion of PTFE/Al-Si energetic mixture, the burning rate of the system, and the high specific heat pulse are significantly higher.

## 4. Conclusions

The research work investigated the preparation of Al-Si-based eutectic alloy powders and described their physicochemical properties, as well as the possibilities of operation in energy mixtures. The main conclusions are as follows:

(1) Al-Si eutectic alloy powder was obtained from economically viable secondary resources and reduced to optimum size by mechanical processing.
(2) The activation energy of Al-Si alloy powders (84.72 kJ/mol) was significantly lower than that of pure aluminum powder (494.36 kJ/mol), because silicon in the alloy lowers the energy required for the initial ignition of jet fuels, and the oxide film on the surface of a pure aluminum particle significantly increases the thermal decomposition temperature. In addition, it can be assumed that scratches, cracks, and deformations during the mechanical grinding of secondary materials reduce the activation energy.
(3) Due to the physicochemical advantages of Al-Si eutectic alloy powder compared to pure aluminum powder, the burning characteristics of PTFE-based energetic mixtures have been improved. In comparison, the burning rate of energetic mixtures prepared with the Al-Si eutectic alloy was 1.5–2.0 mm/s higher than mixtures prepared with pure aluminum.
(4) Oxides, carbides, and fluorides formed when reactive fuels based on Al-Si are burned in energy mixtures are products of high-temperature exothermic chemical reactions. These reactions positively affect the burning kinetics and thermodynamics of energetic mixtures.

## Figures and Tables

**Table 1:** Activation energy of initial materials and energetic mixture.

Reactive Fuels	*β* (Heating Rate, °C/min)	*T_p_*, °C	*E_a_*/(kJ/mol) Kissinger’s Method	*R* ^2^	*E_a_*/(kJ/mol) Ozawa’s Method	*R* ^2^
Al	5	1027.3	494.36	0.91	496.19	0.92
	10	1035.2				
	15	1053.6				
	20	1061.8				
Al-Si	5	904.7	84.72	0.97	105.82	0.98
	10	981.9				
	15	1044.2				
	20	1057.2				
PTFE	5	479.8	81.29	0.94	96.30	0.96
	10	501.6				
	15	527.3				
	20	548.7				
PTFE/Al	5	503.7	156.71	0.93	168.11	0.95
	10	515.8				
	15	529.7				
	20	544.2				
PTFE/Al-Si	5	438.3	61.75	0.88	77.33	0.93
	10	461.7				
	15	483.3				
	20	517.1				

## Data Availability

The data supporting the results can be made available from the corresponding author upon request.
